# The Interplay of RNA Binding Proteins, Oxidative Stress and Mitochondrial Dysfunction in ALS

**DOI:** 10.3390/antiox10040552

**Published:** 2021-04-02

**Authors:** Jasmine Harley, Benjamin E. Clarke, Rickie Patani

**Affiliations:** 1 Department of Neuromuscular Diseases, Queen Square Institute of Neurology, University College London, London WC1N 3BG, UK; jasmine.harley@crick.ac.uk; 2 The Francis Crick Institute, 1 Midland Road, London NW1 1AT, UK; 3 National Hospital for Neurology and Neurosurgery, University College London NHS, London WC1N 3BG, UK

**Keywords:** RNA binding protein, oxidative stress, mitochondrial dysfunction, ALS

## Abstract

RNA binding proteins fulfil a wide number of roles in gene expression. Multiple mechanisms of RNA binding protein dysregulation have been implicated in the pathomechanisms of several neurodegenerative diseases including amyotrophic lateral sclerosis (ALS). Oxidative stress and mitochondrial dysfunction also play important roles in these diseases. In this review, we highlight the mechanistic interplay between RNA binding protein dysregulation, oxidative stress and mitochondrial dysfunction in ALS. We also discuss different potential therapeutic strategies targeting these pathways.

## 1. Introduction

RNA binding proteins (RBPs) represent a family of >1300 proteins that control all aspects of a RNA life cycle, including regulating transcription, processing, localisation, function and finally decay [1,2]. This carefully controlled interaction allows a diligent regulation of gene expression. Dysfunction of RBPs has been heavily implicated in the pathogenesis of many neurodegenerative diseases, in particular amyotrophic lateral sclerosis (ALS). ALS is characterized by upper and lower motor neuron death [3]. Loss of motor neurons results in progressive muscle weakness, paralysis and eventual death, typically due to denervation of respiratory muscles [4]. Up to half of patients also suffer some form of cognitive decline or behavioural impairment, ~15% of which fulfil diagnostic criteria for frontotemporal dementia (FTD) suggesting that ALS and FTD are part of a disease spectrum [5,6]. The study of familial ALS (fALS) cases, which account for ~5–10% of cases overall, has identified ~30 genes involved in pathogenesis [7]. A genetic basis for disease is not restricted to fALS, as mutations in genes found in fALS cases have been identified in up to 25% of apparently sALS cases, which share many neuropathological features [8]. Mutations in genes implicated in ALS encode proteins with diverse cellular functions with some convergence, however, on RBPs including: TAR DNA-binding protein (*TARDBP*), fused in sarcoma (*FUS*), TATA-Box Binding Protein Associated Factor 15 (*TAF15*), EWS RNA Binding Protein 1 (*EWSR1*), heterogeneous nuclear ribonucleoprotein A1 (*HNRNPA1*), heterogeneous nuclear ribonucleoprotein A2/B1 (*HNRNPA2B1*), matrin 3 (*MATR3*), T-cell-restricted intracellular antigen-1 (*TIA1*) and senataxin (*SETX*) (Table 1).

RBP nuclear to cytoplasmic mislocalisation and aggregation is considered a key hallmark of disease, evident across both fALS and sALS cases [9,10,11,12,13,14,15]. Mutations in RBPs can also cause their mislocalisation to the cytoplasm [16,17,18]. Multiple, possibly overlapping, pathomechanisms have been identified in ALS. In addition to RBP dysfunction, increased oxidative stress, mitochondrial dysfunction, impaired nucleocytoplasmic transport, protein dyshomeostasis, deficits in axonal transport, excitotoxicity and non cell autonomous mechanisms of disease arising from glia are thought to contribute to motor neuron death in ALS. Here, we review the dysfunction of RBPs and their interplay with oxidative stress and mitochondrial dysfunction in ALS, as well as highlight potential therapeutic strategies targeting these pathways.

## 2. Mechanisms of RNA Binding Protein Dysfunction in ALS

RBP dysregulation has been proposed to cause ALS pathogenesis through two main mechanisms: an alteration in RNA metabolism and loss of protein homeostasis. Many RBPs autoregulate their own expression [12,13,14,15] and mouse models either knocking down or overexpressing wildtype forms of certain RBPs have been able to partially recapitulate ALS pathological features [29], highlighting the importance of their tightly regulated physiological expression. Due to the diverse roles of RBPs, dysregulation results in a range of molecular phenotypes such as perturbed gene expression, splicing patterns and splicing machinery, mRNA nuclear export, transport and local translation. This could be the result of aberrant protein localisation, aberrant interactions or post-translational modifications, protein misfolding, aggregate formation and changes in granule dynamics. The following sections will discuss how RBP deregulation can in turn disrupt cellular homeostasis.

### 2.1. Localisation

Although ALS is highly genetically heterogeneous and most cases have no clear genetic cause, TDP-43 mislocalisation from the nucleus to the cytoplasm of motor neurons has been found to occur in the vast majority (>95%) of ALS cases [9,30]. Furthermore, nuclear to cytoplasmic FUS [10], TAF15 [22] and SFPQ [14] mislocalisation has been identified in sALS motor neurons. Mislocalisation of TDP-43 [31], SFPQ [14] and FUS [32] has also been reported in mutant VCP-ALS iPSC derived motor neurons. These studies and others provide evidence of perturbed protein subcellular localisation in the absence of overt aggregation. It is still unresolved whether disease is caused by a nuclear loss of function or a cytoplasmic gain in function, and if these events occur simultaneously or sequentially, with the possibility remaining that both events have an equal involvement in disease. Mislocalisation of RBPs may be due to defects in the nucleocytoplasmic machinery [33], modification of the nuclear localisation by mutations or post-translational mechanisms discussed below.

### 2.2. Aggregation

Many proteins implicated in ALS (TDP-43, hnRNPA1, TAF15, EWS, FUS, TIA1) display point mutations in their intrinsically disordered regions (IDRs), which are also called low complexity domains (LCDs). These domains have been shown to be responsible for weak protein-protein interactions, driven by liquid-liquid phase separation (LLPS), allowing the formation of reversible molecular condensates that can assemble/disassemble in response to changes in the cellular environment. Remarkably, over 40 ALS-linked mutations have been found in TDP-43, with only 3 of these not located within the C-terminal LCD [34]. Mutations in the LCD of TDP-43, hnRNPA1, FUS, TIA1, TAF15 and EWS result in increased fibrillar formation of the mutated protein [22,24,27,35,36,37]. This increased fibrillar formation is able to seed prion-like aggregation from the remaining soluble pool of protein, encouraging cytoplasmic aggregation and subsequent nuclear loss. Such seeded aggregation and prion-like behaviour has recently been demonstrated in human iPSC-derived motor neurons and astrocytes, which also exhibited differential vulnerability to recombinant TDP-43 oligomers [38]. In addition, formation of abnormal RNA foci may lead to abnormal RBP mislocalisation. G_4_C_2_ hexanucleotide repeat expansion in the first intron of the *C9ORF72* gene undergoes bidirectional transcription, which then forms RNA foci [39]. These RNA foci have been shown to sequester RBPs, altering their localisation and thus RNA metabolism, including TDP-43, FUS, SFPQ and hnRNPA1 [40,41,42]. It is still a matter of debate as to whether these aggregates contribute to neurodegeneration [43]. The sequestration of proteins could be both protective and detrimental depending on the disease phase.

### 2.3. Aberrant Granule Dynamics

The similarity between cytosolic aggregation of RBPs seen in ALS and the formation of membrane-less granules suggests a role for stress granules (SGs) in ALS pathogenesis. SGs are enriched with proteins containing LCDs, including many ALS-linked RBPs, with mutations in these regions shown to alter SG dynamics and increase aggregation [26,44]. Supporting this, ALS-associated RBPs that have been shown to interact with or alter SG dynamics include, TDP-43 [45,46], FUS [47], hnRNPA1 [27], TIA1 [48] and ATXN2 [49]. As many of these ALS causing mutations result in aberrant phase separation, decreased SG dynamics and persistence, it has been suggested these may develop into the cytoplasmic aggregates found at end stage disease. To support this hypothesis, many SG proteins are found in the cytoplasmic protein aggregates, for example TDP-43 and FUS colocalise with SG markers in the cytoplasmic inclusions found in ALS patient samples [45,50]. However, there is no direct evidence to support this phenomenon and it remains unknown whether SGs function as precursors of the inclusions or if SG components are incorporated into inclusions [9,51,52]. Recently, a collection of studies have advanced our insight into this topic, and shown that upon stress TDP-43 also forms droplets that are distinct from SGs, and which can persist into aggregate-like structures [53,54,55]. This suggests aggregation of TDP-43 and the pathogenic cascade of ALS can occur independently of SGs.

Many RBPs can be found in paraspeckles, nuclear ribonucleoprotein membraneless granules that sequester proteins and RNA to regulate gene expression [56]. Paraspeckles have been hypothesised to have a role in ALS pathology, with ALS-linked RBPs, TDP-43, FUS and SFPQ identified as components of paraspeckles. Whilst these proteins have been shown to be mislocalised from the nucleus to the cytoplasm in ALS motor neurons [9,10,14], other core paraspeckle proteins, NONO and PSPC1 have been shown not to be mislocalised in sporadic ALS postmortem tissue [57]. Furthermore, depletion of FUS or TDP-43 have been shown to decrease paraspeckle formation [58,59]. Increased paraspeckle formation has been found in both mutant FUS and TDP-43 ALS post mortem motor neurons [60,61]. Further supporting data come from identification of an increase in paraspeckle formation in early stage sporadic ALS spinal motor neurons [62].

### 2.4. Post Translational Modifications

Post translational modification (PTM) of RBPs have been shown to tightly regulate their various functions, with aberrant PTMs observed in both fALS and sALS [63]. Phosphorylation of TDP-43 has been linked to aggregation in post mortem ALS tissue [64] but also has been proposed to be a preventive measure to attempt to reduce aggregation [65,66,67,68]. Other modifications to RBPs that affect either RNA binding function or cytoplasmic mislocalisation and/or aggregation include methylation [69,70], sumoylation [71], acetylation [72] and ubiquitination [73]. PTMs of RBPs have been shown to regulate phase separation and SG dynamics with dysregulation in ALS contributing to aggregation and SG dysfunction [70,74,75,76]. Furthermore, RBPs can undergo proteolytic cleavage which generates shorter protein chains, often with modified or new protein activities. Truncated species of TDP-43 can be generated through multiple proteolytic cleavage sites and although the exact function of these proteins remain unclear, they are generally thought to be toxic and are found in aggregates in ALS patients [77,78,79,80,81].

### 2.5. Deregulated RNA Metabolism

RBPs interact with a diverse range of RNAs. As a single RNA binding protein can bind to many thousands of RNA targets, a disturbance in one or more of these RBPs potentially has a broad and diverse impact on RNA metabolism [82,83,84]. Deregulated RNA metabolism has been described at many levels in ALS, including intron retention [14] and skipping of constitutive exons [85,86]. Loss of TDP-43 function has been shown to result in the downregulation of several transcripts including neuronal growth associated protein stathmin-2 [87]. Specific mutations in TDP-43 have been shown to result in additional splicing dysregulation, including gain of function effects [88].

Similarly to TDP-43, FUS has shown to regulate the stability of hundreds of transcripts [89] and many neuronal function-associated molecules have been identified to be regulated by FUS [83,90,91,92,93,94]. Therefore, loss of function of FUS has the potential to have a large impact in gene expression and/or alternative splicing. Both FUS and SFPQ more avidly bind to retained introns and are hypothesised to be transported out of the nucleus by intron-retaining transcripts [14]. Furthermore, ALS-causing mutant FUS has been shown to result in increased intron retention, with many intron retention events occurring in RBPs including FUS itself [15]. Intron retention and enhanced binding of mutant FUS to growth factor BDNF may impair its function [95]. ALS-causing MATR3 mutations have been shown to result in nuclear global mRNA export defects, including the mRNA of TDP-43 and FUS, demonstrating the interconnected network of RBPs and their involvement in ALS [96]. Furthermore, loss of the interaction between SFPQ and FUS has been identified in ALS-FTD [97].

Dysregulated RNA metabolism extends into the cytoplasm and neuronal processes, as RBPs are essential for mRNA transport and translation. TDP-43 ALS-linked mutants have been shown to have disrupted axonal transport dynamics in vitro and in vivo [98,99]. ALS-linked mutant FUS has been shown to drive toxicity through cytoplasmic gain of function effects such as inhibiting local intra-axonal protein translation and suppressing RNAs encoding proteins essential for synaptic function [100]. This study and others provide evidence for the role of RBPs in synaptic activity [101,102].

## 3. RNA Binding Proteins and Oxidative Stress in ALS

Oxidative stress is a process where accumulation of reactive oxygen species (ROS) leads to cellular damage and cell death due to an imbalance between free radical production and antioxidant defences. The presence of high levels of ROS causes damage to several different parts of the cellular machinery through lipid peroxidation and oxidation of proteins and/or DNA. Oxidative stress is thought to increase with age, a major risk factor in ALS [103]. Evidence of oxidative stress has been identified in CSF, plasma, serum and urine of sporadic ALS patients [104,105,106,107] and post-mortem ALS spinal cord tissue [108,109,110,111].

**Figure 1:**
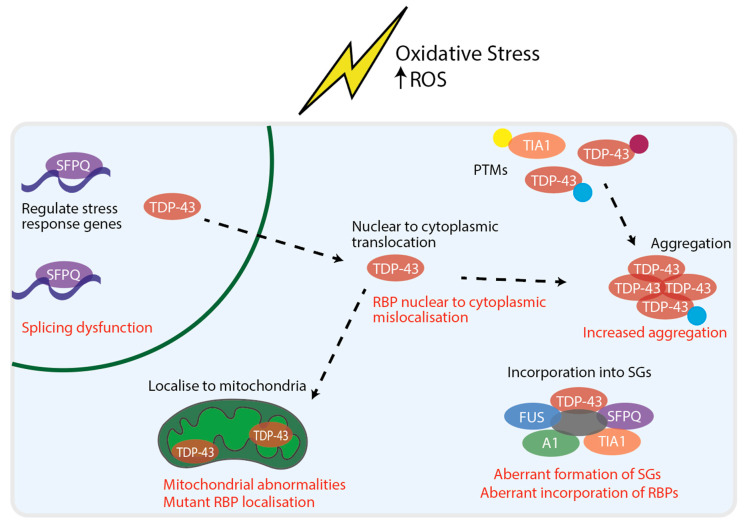
RBP responses to oxidative stress and dysfunction in ALS. RBPs have been shown to respond to oxidative stress by multiple mechanisms. In the nucleus, SFPQ has been shown to regulate stress response genes. TDP-43 has been shown to undergo nuclear to cytoplasmic translocation. In the cytoplasm, oxidative stress has shown to cause PTMs of RBPs (represented by the yellow, magenta and blue dots). PTMs of RBPs have been associated with RBP aggregation. In addition, upon oxidative stress multiple RBPs are incorporated into SGs and TDP-43 has been shown to localise to mitochondria. The RBP response to oxidative stress overlaps with mechanisms of RBP dysfunction that have been implicated in ALS (displayed in red). This includes nuclear to cytoplasmic mislocalisation, aberrant PTMs, increased aggregation, perturbed stress granule dynamics and damage caused by RBP localisation to mitochondria.

Interestingly, links have been made between oxidative stress and RBPs. Oxidative stress has been shown to cause PTMs of RBPs, including cysteine oxidation [112,113] and acetylation [72] of TDP-43, which both led to increased aggregation. Furthermore, oxidative stress induced by multiple experimental methods resulted in TDP-43 phosphorylation, mislocalisation and/or aggregation [114,115,116,117]. TIA1 has also been shown to undergo cysteine oxidation in response to increased ROS production, which subsequently suppressed SG formation and increased apoptosis [118]. Oxidative stress also provokes the recruitment of TDP-43 [119], SFPQ [120], hnRNPA1 [121], TIA1 [48] and ATXN2 [49] to SGs. Thus, oxidative stress driving RBPs to SGs may form a basis for later aggregation. Supporting this, increasing levels of protein oxidation resistance 1 (OXR1) reduced TDP-43 and FUS aggregation [122]. SFPQ has also been implicated in cellular responses to oxidative stress as it has been shown to regulate the transcription of stress response genes and knockdown of *SFPQ* reduced ROS production in sodium arsenite treated cells [123]. Furthermore paraspeckles, in which SFPQ and FUS are core components, have been shown to form upon recovery from arsenite induced oxidative stress [124]. RBP responses to oxidative stress are summarised in Figure 1.

Responses to oxidative stress have been shown to be perturbed in cellular models of ALS. Fibroblasts from ALS patients with *TARDBP* and *C9ORF72* mutations have shown aberrant formation of SG and/or phospho-TDP-43 aggregates in response to chronic oxidative stress [116] and ALS-causing *FUS* mutations have been shown to result in the aberrant incorporation of FUS into SGs [47,125,126,127]. Mutant FUS expression also increases the presence of RBP, ELAVL4, in SGs formed in response to oxidative stress [128]. Furthermore, mutant TDP-43 expressing motor neurons exposed to oxidative stress displayed reduced formation of SGs, dysfunction of vesicle secretion, an altered protein interactome and reduced survival compared with wild type TDP-43 expressing motor neurons [129].

## 4. RNA Binding Proteins and Mitochondrial Dysfunction in ALS

Oxidative stress has been heavily associated with mitochondrial dysfunction in ALS. Mitochondria produce ROS as a by-product of energy production, leading to nitration of mitochondrial proteins, mtDNA damage and dysfunction of the mitochondrial respiratory chain. Abnormal morphology of mitochondria has been reported in sALS post-mortem tissue [130,131] and several ALS-linked mutant proteins have been found to localise to mitochondria in ALS models [132,133,134,135].

Several genes directly involved in mitochondrial function are implicated in ALS pathogenesis including *SIGMAR1* and *CHCHD10* [115,116]. Expression of mutant sigma-1 receptor promoted mitochondrial dysfunction and the mislocalisation of TDP-43 [136]. Interestingly, in this study, supplementation of mutant sigma-1 receptor expressing cells with ATP partially reduced cytoplasmic mislocalisation of TDP-43. Mutations in mitochondrial gene *CHCHD10* have also been linked with ALS-FTD [137,138]. *CHCHD10* knockin mutations resulted in mitochondrial damage and motor neuron degeneration [139]. Knockdown of *CHCHD10* resulted in similar effects on mitochondrial function as expression of mutant *CHCHD10*, suggesting that mutations in *CHCHD10* result in loss of function or dominant negative effects [140]. Interestingly, it was also shown in this study that *CHCHD10* interacted with TDP-43 and mutations or depletion of *CHCHD10* resulted in TDP-43 cytoplasmic mislocalisation in primary hippocampal neurons.

RBPs have important physiological roles in mitochondrial function. TDP-43 interacts with several mitochondrial proteins [141] and has been proposed to be involved in the stabilisation of mitochondrial transcripts [142]. shRNA knockdown of TDP-43 in primary mouse motor neurons reduced the expression of a number of mitochondrial transcripts, mitochondrial number and mitochondrial membrane potential [143]. Meanwhile, overexpression of wild type TDP-43 causes mitochondrial morphological abnormalities [29], affects mitochondrial dynamics [144] and disrupts mitochondrial-ER contacts [145]. Overexpression of wild type FUS also resulted in mitochondrial abnormalities [146]. Furthermore, hnRNPA1 and TIA1 have been shown to regulate mRNAs of proteins involved in mitochondria fission activity, with down regulation of hnRNPA1 and TIA1 both resulting in mitochondrial elongation or increased abundance and overexpression increasing mitochondrial fragmentation [147,148,149]. EWS has also been shown to have a role in mitochondrial and energy homeostasis, with mitochondrial abundance and activity significantly reduced in EWS-deficient mice [150]. Finally, SFPQ is involved in mitochondrial function, as loss of SFPQ caused decreased abundance of mitochondrial oxidative phosphorylation complexes [151] and SFPQ binds to transcripts of mitochondrially localised LaminB2 and Bcl2 adjacent to mitochondria [152].

ALS-linked mutant RBPs have been shown to localise to mitochondria in ALS and cause damage. Mutant TDP-43 has been associated with multiple mechanisms of mitochondrial dysfunction in neurodegeneration (fully reviewed in [153]). In an important recent study, mutant TDP-43 expression resulted in mitochondrial DNA release, which in turn triggered activation of an immune response through the cGAS-STING pathway [154]. TDP-43 has been found to localise to mitochondria in sALS motor neurons [134] and animal models of ALS [134,155]. Mutant TDP-43 localised to mitochondria at higher levels than expression of wild type TDP-43 and caused downregulation of complex I subunits [134,156]. Blocking TDP-43 import into mitochondria prevented complex I dysfunction and neuronal death in mutant TDP-43 mice [134,157]. However, findings of abnormal mitochondrial bioenergetics in mutant TDP-43 mice and patient fibroblasts could not be confirmed by another group [158]. Truncated forms of TDP-43 have been found to have a higher propensity to accumulate in the intermembrane space of mitochondria [159]. Interestingly, these truncated forms of TDP-43 were less damaging to mitochondria than full length mutant TDP-43, possibly due to the lack of truncated forms in the mitochondrial matrix.

In addition to TDP-43, mutant FUS has also caused damage to mitochondria [133]. Overexpression of mutant FUS led to increased association with mitochondrial transcripts and decreased mitochondrial membrane potential and respiration through a gain in toxic function mechanism [160]. Furthermore, an alternative open reading frame leads to another disease relevant form of FUS, which when overexpressed led to mitochondrial dysfunction [161]. Recent transcriptomic analysis of a FUS mutant mouse has also revealed dysregulation of mitochondrial transcripts [162]. In a more physiological knockin model of humanised mutant FUS, mislocalisation of FUS coincided with downregulated mitochondrial transcripts [163]. Furthermore, transgenic flies with ALS linked mutations in TDP-43, FUS and TAF15 showed mitochondrial fission defects in muscle and motor neurons [164]. Interestingly, this mitochondrial fragmentation could be rescued by the expression of dominant negative mutant form of DRP1, a protein responsible for coordinating mitochondrial dynamics, shown to be regulated by hnRNPA1 [147].

## 5. Targeting RNA Binding Proteins, Oxidative Stress and Mitochondrial Dysfunction in ALS

Currently there are only two FDA approved drugs for ALS: riluzole and edaravone, which both only have modest effects on disease progression. Riluzole, a glutamatergic neurotransmitter inhibitor which may reduce oxidative stress [165,166], only extends life (or time to tracheostomy) by 3 months [167]. Edaravone (approved in Japan, Korea, US, Canada and Switzerland), is an antioxidant drug in which the exact mechanism of action remains unknown and has been shown to cause a modest reduction in progression in early stage disease [168].

Many small molecules have been shown to reduce the cytoplasmic accumulation and aggregation of TDP-43 and FUS, targeting autophagy and SG pathways [169,170,171]. Small molecules have also been identified to target the RBPs themselves. The RNA recognition motif 1 (RRM1) domain of TDP-43 has shown to be a druggable site, with a small molecule able to reduce RNA binding and improve neuromuscular strength in an ALS drosophila model [172]. Supporting this as a possible therapeutic target, an antibody targeting the RRM1 domain has shown to reduce TDP-43 proteinopathy, cognitive impairment, motor defects and neuroinflammation in a TDP-43 ALS mouse model [173]. Targeting RBPs extends beyond TDP-43, with an ASO targeting ataxin-2 shown to reduce TDP-43 aggregation, improve motor function and improve survival in a TDP-43 transgenic mouse model [174].

Targeting RBP localisation to mitochondria may also be a potentially effective therapeutic target. Peptides designed to block the mitochondrial entry of TDP-43 prevented mitochondrial dysfunction and rescued motor neuron death, neuromuscular junction denervation and motor dysfunction in a mutant TDP-43 overexpressing mouse [134,157].

A number of therapies targeting mitochondria and/or oxidative stress have provided benefits in animal models but have performed disappointingly in clinical trials, such as Vitamin E [175,176], N-acetyl-L-cysteine (NAC) [177,178], Coenzyme Q10 [179,180] and Dexpramipexole (RPPX) [181,182]. This may highlight the lack of translatability and overreliance on animal models, especially the mutant SOD1 mouse model. Despite these failures there are a few ongoing clinical trials that are testing antioxidants. This includes a phase II clinical trial for a compound that targets NAD+, a coenzyme involved in redox reactions in cells, accompanied with the use of over the counter antioxidants (NCT04244630).

## 6. Conclusions

In this review, we have summarised potential mechanisms of RBP dysfunction in ALS and reviewed the emerging link between RBP dysfunction and both oxidative stress and mitochondrial dysfunction in this context. Although significant overlap has been observed in these pathomechanisms, it is likely that polytherapy targeting multiple pathomechanisms will be required to elicit more efficacious therapeutic benefits. It is important to consider that targeting certain pathways may be more responsive depending on the particular disease phase and thus it is important to resolve the primacy of molecular and cellular pathological events. Further work dissecting these mechanisms may guide the development of new therapies, which are desperately required in this relentlessly progressive and invariably fatal disease.

## Figures and Tables

**Table 1:** Genes encoding RBPs involved in ALS.

Gene fALS	Cases (%)	Mutations	References
TARDBP	5	>40	[19]
FUS	4	>40	[20,21]
TAF15	<1	<10	[22,23]
EWSR1	<1	<10	[24]
SETX	<1	<10	[25]
TIA1	<1	<10	[26]
HNRNPA1	<1	<5	[27]
HNRNPA2/B1	<1	<5	[27]
MATR3	<1	<5	[28]

## Data Availability

Data sharing not applicable.
